# Toward a Network Model of MHC Class II-Restricted Antigen Processing

**DOI:** 10.3389/fimmu.2013.00464

**Published:** 2013-12-16

**Authors:** Michael A. Miller, Asha Purnima V. Ganesan, Laurence C. Eisenlohr

**Affiliations:** ^1^Department of Microbiology and Immunology, Thomas Jefferson University, Philadelphia, PA, USA

**Keywords:** antigen processing, antigen presentation, MHC class II, alternative processing, endogenous, review

## Abstract

The standard model of Major Histocompatibility Complex class II (MHCII)-restricted antigen processing depicts a straightforward, linear pathway: internalized antigens are converted into peptides that load in a chaperone dependent manner onto nascent MHCII in the late endosome, the complexes subsequently trafficking to the cell surface for recognition by CD4^+^ T cells (T_CD4+_). Several variations on this theme, both moderate and radical, have come to light but these alternatives have remained peripheral, the conventional pathway generally presumed to be the primary driver of T_CD4+_ responses. Here we continue to press for the conceptual repositioning of these alternatives toward the center while proposing that MHCII processing be thought of less in terms of discrete pathways and more in terms of a network whose major and minor conduits are variable depending upon many factors, including the epitope, the nature of the antigen, the source of the antigen, and the identity of the antigen-presenting cell.

## The Classical Pathway Takes Shape

**Figure 1:**
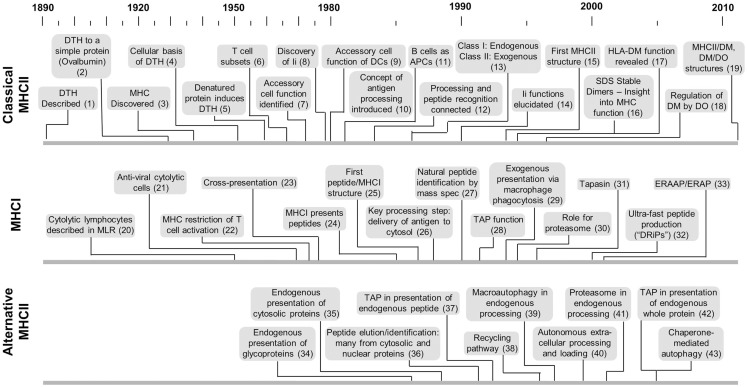
**Timelines of key developments with respect to MHCII-classical processing, MHCI processing, and MHCII-alternative processing**. This is not intended to provide a comprehensive listing but more a sense of the genesis and evolution of each area with respect to the others. Citation key: **(1)** Koch (117), **(2)** Dienes and Schoenheit (118), **(3)** MHC discovered by Gorer (119), **(4)** Gell and Hinde (120), **(5)** Gell and Benacerraf (2), **(6)** Claman et al. (121), **(7)** Shevach and Rosenthal (5), Rosenthal and Shevach (122), **(8)** Jones et al. (123), **(9)** Nussenzweig (124), Steinman and Nussenzweig (125) after earlier identification by Steinman and Cohn (126) and later demonstration of antigen processing by Sunshine et al. (23), Van Voorhis et al. (24), **(10)** Ziegler and Unanue (8), **(11)** Chesnut and Grey (20), Chesnut et al. (99, 100), Lanzavecchia (21), **(12)** Shimonkevitz et al. (11), Babbitt et al. (13), **(13)** Germain (54), **(14)** Bakke and Dobberstein (27), Peterson and Miller (29), Roche and Cresswell (30), Teyton et al. (31), Roche et al. (32), **(15)** Brown et al. (127), **(16)** Germain and Hendrix (35), Sadegh-Nasseri and Germain (36), Amigorena et al. (37), Qui et al. (38), Tulp et al. (39), West et al. (40), Riberdy et al. (128), **(17)** Fling et al. (25), Morris et al. (26), **(18)** Denzin et al. (129), **(19)** Pos et al. (130), Guce et al. (131), **(20)** Govaerts (132), **(21)** Lundstedt (133), **(22)** Zinkernagel and Doherty (134), **(23)** Bevan (55), **(24)** Townsend et al. (135), **(25)** Bjorkman et al. (136), **(26)** Moore et al. (47), Yewdell et al. (48), **(27)** Falk et al. (137), Rötzschke et al. (138), Van Bleek and Nathenson (139), **(28)** Powis et al. (49), **(29)** Kovacsovics-Bankowski et al. (140), **(30)** Rock et al. (52), **(31)** Sadasivan et al. (141), **(32)** Reits et al. (142), Schubert et al. (143), **(33)** Brouwenstijn et al. (144), Serwold et al. (145), **(34)** Bikoff and Bershtein (75), Eisenlohr and Hackett (77), Weiss and Bogen (78), **(35)** Jacobson et al. (81, 82), **(36)** Rudensky et al. (87), **(37)** Malnati et al. (83), **(38)** Pinet et al. (70), **(39)** Brazil et al. (146), **(40)** Santambrogio et al. (62, 63), **(41)** Mukherjee et al. (92), **(42)** Tewari et al. (94), **(43)** Zhou et al. (147).

In the early twentieth century delayed-type hypersensitivity (DTH) established itself as the mainstay for cellular immunologists, providing the launching point for many of the antigen systems that remain in use today (1) (Figure 1). A point of emphasis is that many of these proteins (ovalbumin, lysozyme, myoglobin, …) shared the properties of being plentiful and sturdy, and therefore amenable to the early protein purification schemes, which were relatively harsh and inefficient. These properties also facilitated structure determination, reinforcing their popularity as immunologists sought greater mechanistic insight into immune recognition.

By the mid-1970s it was known that denatured proteins and synthetic peptides could induce DTH (2, 3) and that DTH is mediated by the “helper” T cell subset (4). This foundation provided the springboard for two key subsequent discoveries. First, the Rosenthal laboratory demonstrated that *in vitro* T cell activation requires an MHCII-compatible “accessory cell,” later renamed antigen-presenting cell (APC) (5–7). At the time, the accessory cell was synonymous with the macrophage, whose longstanding reputation for phagocytosis further reinforced focus on exogenously provided antigens. Dendritic cells (DCs) and B cells would only later be identified as “professional” APC (Figure 1). Subsequently Unanue and co-workers pioneered the concept of “processing” in which antigen is taken up by the APC and handled internally for a defined period of time before emerging on the cell surface in a form capable of activating T_CD4+_ (8). A fragmentation step was implied by the observation that presentation is inhibitable by weak bases that prevent activation of the endosomal proteases (9).

While *Listeria monocytogenes* was utilized in initial experiments (8, 10), the nominal DTH antigens were far more suitable for experiments that sought greater mechanistic insight, due in large part to the challenge of epitope identification. At the time, the standard approach entailed chemical and/or proteolytic fragmentation of whole antigen, identification of the active fragment with *in vitro* assays, and confirmation and fine mapping with synthetic peptides. Thus, it was demonstrated with the ovalbumin system that the same T_CD4+_ hybridoma could be activated by whole antigen or proteolytic OVA fragments provided to the APC, or synthetic peptide pulsed onto gluteraldehyde-fixed APCs (11). An epitope within hen egg lysozyme (12) was used to demonstrate that the immunogenic peptide binds directly to MHCII (13) and, later, to map the residues of the peptide that contact MHCII and those that contact the T cell receptor (14). Subsequent key insights were made using the same or similar globular protein antigen systems, including the identification of specific endosomal proteases that participate in antigen processing (15–19), the impact that surface immunoglobulin has on the efficiency and specificity of processing by B cells (20–22), the antigen processing abilities of DCs (23, 24), and the critical role that the chaperone HLA-DM (in humans, H2-M in mice, referred to collectively as “DM”) plays in late endosomal (“classical”) peptide loading (25, 26).

Concurrent biochemical experiments served to reinforce the classical pathway. Efforts by several groups, most notably the Cresswell laboratory, elucidated the role of the transient MHCII binding partner, invariant chain (Ii), in delivering MHCII to the late endosome where Ii is removed by the combined actions of proteases and DM, and high affinity peptides are loaded (27–34). Germain and co-workers subsequently demonstrated that acquisition of high affinity peptide correlates with a discernible change in MHCII conformation, the so-called SDS-resistant “compact dimer” (35, 36). Several groups exploited this property to demonstrate via subcellular fractionation that compact dimer formation occurs in the late endosome (37–40), and is generally dependent upon DM (41, 42). Direct imaging studies tracing the fates of MHCII and Ii generally provided corroboration (43–46).

## MHC Class I Processing: Fading Contrast

The processing of antigen for recognition by CD8^+^ T cells (T_CD8+_) had long been viewed as fundamentally different. This is because most MHC class I (MHCI) processing begins with delivery of antigen to the cytosol (47, 48), usually via infection, which allows for access to the proteasome and the transporter associated with antigen processing (TAP), both being critical for the production and delivery of most peptides to nascent MHCI in the ER (49–52). The apparent dichotomy – MHCI for endogenous antigen and MHCII from exogenous antigen – was reinforced by Morrison et al. who reported that inactivation of influenza virus obviates T_CD8+_ but not T_CD4+_ cell line activation while expression of influenza protein by a recombinant vaccinia virus results in T_CD8+_ but not T_CD4+_ activation (53). Thus, MHCI and MHCII appeared to be fundamentally different in terms of where the peptides come from (54).

The distinction stood for many years until the concept of cross-presentation, essentially MHCI-restricted presentation of exogenous antigen, gained traction. First observed by Bevan as the development of a host response to allogeneic cells (55), cross-presentation was eventually demonstrated to apply to virus infection, and attributed in most cases to the ability of the DC to take up material released from the antigen bearing cell and transfer it to the cytosol for conventional processing via mechanisms that are still under investigation (56).

## Alternative MHCII Processing

Cross-presentation expands the potential for T_CD8+_ activation in two ways. First, it ensures delivery of antigen to DCs, generally considered essential for T cell priming (57), under circumstances when the invading organism does not infect DCs. Second, it short circuits many of the strategies that pathogens have developed to thwart MHCI antigen processing (58). For the similar purpose of expanding the potential for T_CD4+_ activation, one might expect that additional mechanisms exist for the generation of MHCII-peptide complexes. Indeed, there is longstanding evidence for alternative MHCII processing pathways, although they have yet to take hold like cross-presentation. These pathways fall into three general categories.

### Extracellular processing and loading

Exceptions to the need for intracellular antigen processing were provided by reports that location of the epitope within a disordered region of the antigen (59), denaturation of the antigen (60), and catabolism of some antigens by serum proteases (61), precludes the need for internalization. Subsequently it was demonstrated that immature DCs secrete lysosomal proteases into the extracellular space and express abundant amounts of empty (peptide receptive) MHC, allowing for autonomous extracellular processing (62, 63) and peptide loading at the cell surface independent of DM (64–66). More recently, extracellular processing of B cell receptor-captured antigen has been demonstrated to occur within the synaptic space between B cell and T_CD4+_ (67). Despite providing a potential explanation for the presentation of antigens that preclude internalization (large parasites, for example), extracellular processing has thus far been vastly understudied.

### The recycling pathway

Experiments demonstrating an active peptide exchange mechanism in live APCs at relatively neutral pH (68) and the ability of mature MHCII to be internalized (69) suggested that MHCII could be recycled for a second round of peptide loading in an early endosomal compartment. Functional operation of this pathway was first demonstrated by the laboratory of Eric Long (70), and subsequently demonstrated to operate independent of DM (71). Influenza hemagglutinin, the antigen they focused upon, represents a group of proteins, including other viral glycoproteins and many bacterial toxins, that may be particularly relevant to the recycling pathway as they unfold in the early endosome as part of their biological programs (72–74).

### Endogenous pathways

Beginning in the mid-1980s, reports emerged that efficient, and in some cases any detectable presentation of a specific MHCII epitope depends upon synthesis of the antigen within the APC (75–80). Because the first antigens were glycoproteins, the suspicion remained, despite many controls, that the antigens trafficked in some fashion to the endosomal compartment for conventional processing. This concern was obviated by the Long lab, who demonstrated efficient presentation of epitopes within cytosolically located measles virus matrix protein and signal sequence-deleted influenza hemagglutinin (81–83). Further, expression of matrix by a recombinant vaccinia virus, where the antigen is not incorporated into the virion, also resulted in presentation (84). Of note, presentation from cytosolic HA was independent of TAP expression, but presentation from a cytosolic “minigene” construct was TAP-dependent (83).

Numerous subsequent reports have provided additional evidence of endogenous presentation (85, 86), including demonstrations that a substantial portion of peptides eluted from MHCII are derived from cytosolic and nuclear proteins (87, 88). Particularly notable is the variety of underlying mechanisms compared to the classical pathway. The Münz laboratory has focused upon a role for macroautophagy, the process in which cytosolic contents are enveloped in a bilayer membrane that subsequently fuses with the lysosome (89). The Blum laboratory has demonstrated that chaperone mediated autophagy, which involves translocation of individual proteins bearing the KFERQ motif, is critical in the presentation of some self-antigens (90). A third form of autophagy, microautophagy, in which cytosolic proteins are delivered to the late endosome during multivesicular body formation (91), is also likely to contribute to MHCII processing although a direct connection remains to be made. We and others have shown roles for the proteasome (92–95) and even TAP (94) in endogenous processing of some proteins. Like cross-presentation, these observations blur the line between the MHCI and MHCII systems.

## At a Crossroads?

Several key questions concerning MHCII processing remain.

(1) How common are the alternative pathways? As recounted elsewhere (86), our laboratory has addressed this question with influenza and ectromelia viruses, and results suggest that most of the T_CD4+_ responses to both viruses is driven by alternative, mainly endogenous processing. It will be of great interest to determine the extent to which this holds for other viruses and other types of pathogens.
(2) Where are MHCII molecules loaded in the cell? An intriguing alternative to the endosomal compartment is the endoplasmic reticulum, the site of Ii loading. There is no obvious reason why incompletely folded proteins could not compete with Ii. Indeed, there are longstanding reports of ER-resident MHCII binding proteins other than Ii (96). The greater challenge has been to connect this event with T_CD4+_ activation. This is exacerbated by the extreme sensitivity of T cells, allowing for the possibility that ER loading is a far greater contributor to T_CD4+_ activation than is appreciable by biochemical assays. Compared to the endocytic compartment there may be limited competition for MHCII in the ER, particularly during a virus infection that shuts off host synthesis. Thus, the bulk of MHCII may be directed to the endosomal compartment due to less efficient acquisition of epitopes derived from exogenous antigen.
(3) How many components of the antigen processing machinery remain to be discovered? Probably many. Components of any metabolic pathway are generally not appreciable until they are disabled, often unintentionally. For example, the role of DM in MHCII processing was revealed by a mutagenesis screen intended to identify MHCII structural mutants (97). A recent antibody-based genome-wide siRNA screen by the Neefjes lab revealed 276 genes that contribute to peptide presentation, only 10% of which had been previously implicated (98). The screen was performed on uninfected cells, and it is possible that infection and the ensuing innate activation enlists additional cellular components.

## Additional Considerations

The data in aggregate indicate that MHCII processing and presentation extends well beyond the classical pathway. Following are additional considerations leading up to our proposal for a different way of thinking about MHCII processing:

(1) A cellular component can contribute to different processing schemes. For example, H-2M can contribute to loading of both proteasome-dependent and -independent epitopes (94).
(2) The same epitope can be produced via multiple pathways. We were first made aware of this possibility by an epitope within the influenza hemagglutinin that is presentable from exogenous antigen via the recycling pathway and from endogenous sources by a proteasome-dependent pathway (73, 94). Since that time we have expanded the analysis, finding that most of the influenza epitopes we have examined, are presentable from both exogenous and endogenous sources of the parent antigen, just much more efficiently in most cases via endogenous sources (unpublished). We speculate that an important factor is the proteolytic activity of various subcellular compartments (the cytosol being relatively hospitable and the endosomal compartment being relatively inhospitable) and the resistance of particular linear sequences to attack by resident proteases.
(3) Different APC types have different antigen processing capabilities. This has been apparent since the first comparisons of DCs, macrophages and B cells (99–104) and has been attributed to a variety of factors including internalization capabilities, protease profile, and activation state (105–112). We recently compared the abilities of three distinct primary APC types (bone marrow-derived DCs, splenic DCs, and peritoneal macrophages) to present six different influenza epitopes from exogenous and endogenous sources. We observed clear differences that were more or less accentuated depending upon the epitope (unpublished observation). An important ramification is that the presentation characteristics of APCs *in vitro* will not necessarily reflect antigen presentation *in vivo*, which in many cases may be an aggregate of several APC types.
(4) Different pathogens will likely demonstrate different processing signatures, both in terms of the processing pathways that are utilized and the extent to which each one is utilized. The various categories of pathogen interact with APCs in profoundly different ways. Large parasites, such as helminths, are presumably processed exogenously, via either the classical or extracellular pathways following some degree of extracellular digestion. This may also be the case for extracellular bacteria although, as noted above, membrane crossing bacterial toxins may be an exception. Viral proteins will have access to endogenous pathways but pathway usage may also be dramatically different depending upon replication scheme, the extent to which vesicular trafficking is disrupted, and the selective activation of cellular processes (e.g., autophagy).
(5) MHCII processing diversity – facilitation and benefits. The open-ended nature of the MHCII-peptide binding groove ensures that many epitope-containing forms can be presented. In contrast, closed-ended MHCI molecules generally require peptides of specific lengths and, thus, more focused and coordinated processing events. A driving force for the existence of multiple pathways is likely the greater diversity of peptides displayed, thereby ensuring sufficient T_CD4+_ engagement, whose importance in resolving infections is becoming increasingly apparent (113–116). In addition, processing diversity reduces the prospect that MHCII-restricted antigen presentation can be thwarted by an infectious agent. This redundancy may be one reason why there are many more reports of pathogens that attack components of the class I vs. class II processing machinery.

## A Network Model of MHCII Antigen Processing

**Figure 2:**
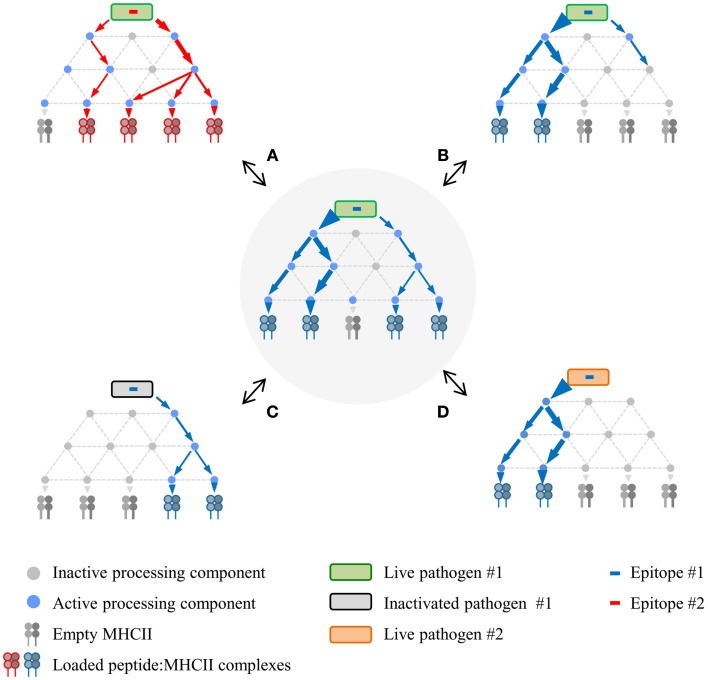
**Proposed network model of MHCII antigen processing**. The network in the center represents a hypothetical processing scheme for epitope #1 derived from live pathogen #1 following infection of APC #1. Processing variations surround this central network, demonstrating altered processing and presentation depending on different epitopes from the same live pathogen **(A)**, different APC for the same epitope **(B)**, same epitope from inactivated pathogen **(C)**, or same epitope expressed via recombination by a different live pathogen **(D)**. Processing components can be either active or inactive depending on APC type and/or APC activation state in response to infection.

The physical properties of the DTH proteins considerably narrow processing scenarios, resulting in the detailed mechanistic insights that have been gained. In contrast, the robust diversity in alternative MHCll processing leads to superficially conflicting data and a diffusion of effort, both of which impede validation by concerted mechanistic studies. A way forward might be facilitated by reframing the problem. Therefore, based upon the points that have been made, we propose a network model of MHCII antigen processing (Figure 2), in which each epitope is produced via several pathways, the composition and balance of which are unique for each epitope, and strongly modulated by factors such as APC type and nature of the infectious agent. *In vitro* experimentation may validate the general tenets of the model, but its true scope will only be appreciated *in vivo*, a far more complex landscape that will be tackled only with the development of additional tools. This framework could catalyze insight into host/pathogen interplay and, consequently, new strategies for rational vaccine design. A critical first step will be increased numbers of investigators who use infectious organisms to investigate MHCII antigen processing and presentation. The systems are admittedly more cumbersome but the territory that they open up more than compensate for the extra effort.

## Author Contributions

Laurence C. Eisenlohr wrote the first draft. Laurence C. Eisenlohr and Michael A. Miller worked on revisions and the figures together.

## Conflict of Interest Statement

The authors declare that the research was conducted in the absence of any commercial or financial relationships that could be construed as a potential conflict of interest.
